# Evaluation of Arterial Stiffness for Predicting Future Cardiovascular Events in Patients with ST Segment Elevation and Non-ST Segment Elevation Myocardial Infarction

**DOI:** 10.1155/2013/792693

**Published:** 2013-10-30

**Authors:** Oguz Akkus, Durmus Yildiray Sahin, Abdi Bozkurt, Kamil Nas, Kazım Serhan Ozcan, Miklós Illyés, Ferenc Molnár, Serafettin Demir, Mücahit Tüfenk, Esmeray Acarturk

**Affiliations:** ^1^Sanliurfa Siverek State Hospital, 63600 Sanliurfa, Turkey; ^2^Department of Cardiology, Adana Numune Training and Research Hospital, Adana, Turkey; ^3^Department of Cardiology, Faculty of Medicine, Cukurova University, Adana, Turkey; ^4^Department of Radiology, Szent János Hospital, Budapest, Hungary; ^5^Heart Institute, Faculty of Medicine, University of Pécs, Pécs, Hungary; ^6^Department of Hydrodynamic Systems, Budapest University of Technology and Economics, Budapest, Hungary; ^7^Department of Cardiology, Adana State Hospital, Adana, Turkey

## Abstract

*Background*. Arterial stiffness parameters in patients who experienced MACE after acute MI have not been studied sufficiently. We investigated arterial stiffness parameters in patients with ST segment elevation (STEMI) and non-ST segment elevation myocardial infarction (NSTEMI). *Methods*. Ninety-four patients with acute MI (45 STEMI and 49 NSTEMI) were included in the study. Arterial stiffness was assessed noninvasively by using TensioMed Arteriograph. *Results*. Arterial stiffness parameters were found to be higher in NSTEMI group but did not achieve statistical significance apart from pulse pressure (*P* = 0.007). There was no significant difference at MACE rates between two groups. Pulse pressure and heart rate were also significantly higher in MACE observed group. Aortic pulse wave velocity (PWV), aortic augmentation index (AI), systolic area index (SAI), heart rate, and pulse pressure were higher; ejection fraction, the return time (RT), diastolic reflex area (DRA), and diastolic area index (DAI) were significantly lower in patients with major cardiovascular events. However, PWV, heart rate, and ejection fraction were independent indicators at development of MACE. *Conclusions*. Parameters of arterial stiffness and MACE rates were similar in patients with STEMI and NSTEMI in one year followup. The independent prognostic indicator aortic PWV may be an easy and reliable method for determining the risk of future events in patients hospitalized with acute MI.

## 1. Introduction

Acute myocardial infarction (AMI) continues a worldwide cause of mortality [1]. In-hospital and 6-month-mortality are approximately 5–7% versus 12-13%, respectively [2, 3]. Estimated risk of mortality for AMI is based on the clinical status of the patients [4]. Recent studies showed that conventional risk factors are inadequate for predicting cardiovascular (CV) mortality and morbidity. A novel risk factor called arterial stiffness, which is a defined reduction of the compliance of arterial wall, and relationship between coronary heart disease (CHD) have been demonstrated. Arterial stiffness results in faster reflection of the forward pulse wave from bifurcation points in peripheral vessels. As a result of new waveform, systolic blood pressure (SBP) increases, diastolic blood pressure (DBP) decreases, cardiac workload increases, and coronary perfusion falls down. It plays a major role in the determination of cardiovascular outcomes, and it is not inferior to the traditional risk factors to assess the future risk [5, 6]. Elevated arterial stiffness is associated with increased major adverse cardiovascular events (MACE) such as unstable angina, AMI, coronary revascularization, heart failure, stroke, and death [7]. Arterial stiffness parameters including mean arterial pressure (MAP), pulse pressure (PP), PWV (m/s), and augmentation index (AI) are directly proportional to the risk of MACE [8–10].

PWV is a susceptible diagnostic element, and it is also involved in risk stratification for subclinical organ damages [11]. Few studies regarding arterial stiffness demonstrated that PWV exhibits a close effect with coronary heart disease [5, 12, 13]. Whether arterial stiffness parameters are related to MACE after acute MI has not been studied sufficiently. The aim of our study was to compare arterial stiffness parameters in patients with ST segment elevation (STEMI) and non-ST segment elevation myocardial infarction (NSTEMI) and to validate its prognostic value.

## 2. Patients

 Ninety-four patients with acute MI (72 men and 22 women, mean age 60,41 ± 11,17) were included in the study. There were 45 STEMI and 49 NSTEMI. Data of patients were analyzed within 24 hours after hospitalization. All patients received eligible treatment according to ESC guidelines. The choice of preparations was entrusted to the investigator. Hemodynamically compromised patients (Killip classifications II, III, and IV), patients with chronic atrial fibrillation and/or flutter, chronic renal failure, mild-severe valvular heart diseases and other chronic diseases were excluded. Our local ethics committee approved the study, and written informed consent was obtained from all participants. Patients were followed up for 12 months. 

## 3. Diagnosis of Acute Myocardial Infarction

Diagnosis of AMI was based on symptoms, elevated cardiac markers, and electrocardiogram (ECG) changes. Patients with typical chest pain plus ECG changes indicative of an AMI (pathologic Q waves, at least 1 mm ST segment elevation in any 2 or more contiguous limb leads or new left bundle branch block, or new persistent ST segment and T wave changes diagnostic of a non-Q wave myocardial infarction) or a plasma level of cardiac troponin-T level above normal.

## 4. Laboratory Findings

 Troponin T, creatine kinase-MB fraction (CK-MB), serum urea, creatinine, eGFR, and other hematological parameters were checked at the admission. 

 Risk factors, such as hypertension, hyperlipidemia, diabetes mellitus, cigarette smoking, and family history, were recorded. Hypertension was considered as SBP and DBP greater than 140 mmHg and 90 mmHg, respectively, using an antihypertensive medication. Diabetes mellitus, hyperlipidemia, and hypertriglyceridemia were defined as using antidiabetic drugs or fasting blood glucose over 126 mg/dL, as plasma low-density lipoprotein cholesterol (LDL-C) >130 mg/dL, using lipid-lowering drugs at the time of investigation, and as TG level >150 mg/dL, respectively, according to the Third Report of the National Cholesterol Education Program guidelines. First-degree relatives who are exposed to coronary artery disease (CAD) before the age for male is <55 and female <65 were considered as family history.

## 5. Pulse Waveform Analysis

Assessment of arterial stiffness was performed noninvasively with the commercially available TensioMed Arteriograph. We collected the oscillometric pulse waves from the patients. We measured the distance between the jugulum-symphysis (which is equal to the distance between the aortic root and the aortic bifurcation), and PWV was calculated. Pulse waves were recorded at suprasystolic pressure. The oscillation signs were identified from the cuff inflated at least >35 mmHg above the systolic blood pressure. In this state there was a complete brachial artery occlusion, and it functions as a membrane before the cuff. Pulse waves hit the membrane, and oscillometric waves were measured by the device and we could see the waveforms on the monitor. The AI was defined as the ratio of the difference between the second (P2 appearing because of the reflection of the first pulse wave) and first systolic peaks (P1 induced by the heart systole) to pulse pressure (PP), and it was expressed as a percentage of the ratio (AI = [P2 − P1]/PP × 100). SBP, DBP, PP, and heart rate and other hemodynamic parameters as return time (RT in sec.), diastolic reflection area (DRA), systolic area index (SAI %), and diastolic area index (DAI %) were measured noninvasively. DRA reflects the quality of the coronary arterial diastolic filling (SAI and DAI are the areas of systolic and diastolic portions under the pulse wave curve of a complete cardiac cycle, resp.). Hence, the bigger the DAI and DRA are, the better the coronary perfusion is. Furthermore, RT is the PWV time from the aortic root until the bifurcation and return, so this value is smaller as the aortic wall is stiffer.

## 6. Followup

The patients were followed up approximately 30 to 330 days (mean, 131 ± 115 d) for the occurrence of MACE.

## 7. Statistical Analysis

Statistical analysis was performed using SPSS 18.0 software package. Categorical measures were summarized as number and percentage and numerical measures as mean and standard deviation (wherever necessary, the median and the minimum-maximum). Chi-square test was used to compare categorical measures between the groups. The quantitative measurements of independent groups were compared by either *t*-test or Mann Whitney *U* test. If the assumptions were ensured we use *t*-test, if failure to provide the assumptions in the cases were ensured, we used Mann Whitney *U* test. We utilized logistic regression analysis to determine the risk factors and criteria for positivity of MACE. Logistic regression analysis was used to determine the variables that affect positivity of MACE and the odds ratios (OR) were obtained. Receiver operator characteristic (ROC) curve analysis was performed to identify the optimal cutoff points for statistically significant numerical measures (at which sensitivity and specificity would be maximal) for the prediction of MACE. *P* value of ≤0.05 was considered significant.

## 8. Results

Ninety-nine patients were examined. Two patients who died in hospital after the first admission and 2 patients whom due to inability to gain information during the followup were excluded. One patient who died during followup because of new onset lung cancer was excluded. Ninety-four of them were found to be eligible (72 men and 22 women, mean age 60,41 ± 11,17) and included in the study. There were 45 STEMI and 49 NSTEMI. Participants were separated into two groups based on MACE observed subjects (Group I) and event-free subjects (Group II). We observed 15 (16%) patients with MACE during 12 months; 79 (84%) patients did not experience any acute event. There was no significant difference at MACE rates between STEMI and NSTEMI patients. Arterial stiffness parameters were found to be higher in NSTEMI group but did not achieve statistical significance apart from pulse pressure (*P* = 0,007).  Table 1 shows the variables between the types of MI patients. Pulse pressure and heart rate were significantly higher in MACE observed group (*P* = 0,012 and *P* = 0.024, resp.). Aortic pulse wave velocity (PWV), AIaortic, systolic area index (SAI), heart rate, and pulse pressure were found to be higher, and ejection fraction, the return time (RT), diastolic reflex area (DRA), and diastolic area index (DAI) were found to be significantly lower in patients with major cardiovascular events.  Table 2 shows clinical, laboratory, and hemodynamic characteristics of the study population.

High density lipoprotein cholesterol (HDL-C) was found to be lower in Group I compared to Group II (*P* = 0.002); other biochemical parameters were not significantly different. Ejection fraction was lower in Group I at first admission.

Group I patients had a higher PWV and smaller RT compared to the Group II (*P* < 0.001). Pulse pressure (*P* = 0.012) and heart rate per minute (*P* = 0.024) were found to be distinctly different between groups. Diastolic area index and DRA were significantly lower in Group I compared to Group II (*P* = 0.034 and *P* = 0.013, resp.). The difference of AI had a significance (*P* = 0.033) between groups. However, PWV, heart rate, and ejection fraction were found to be independent indicators at development of MACE (resp., *P* ≤ 0.001, *P* = 0.036, and *P* = 0.047) after logistic regression analysis. Every one m/s increase in PWV caused 2.566 times increase in odds ratio (OR) (95% confidence interval (CI), 1.447 to 4.551; *P* < 0.001) for MACE development and caused a positive association between one beat/min increase in heart rate and MACE (OR = 1.080, 95% CI, 1.006 to 1.185; *P* = 0.036) for the development of cardiovascular events. Odds ratio for ejection fraction was 0.907 (95% CI, 0.823 to 0.999; *P* = 0.047). 

**Figure 1:**
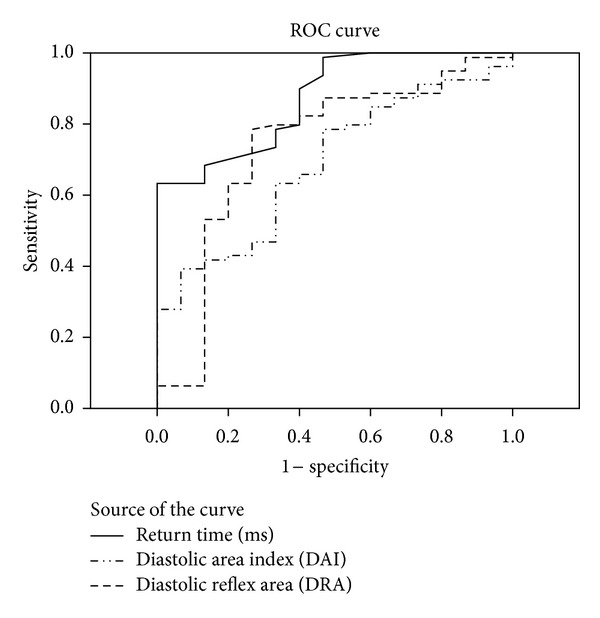
It shows sensitivity and specificity of the parameters.

**Figure 2:**
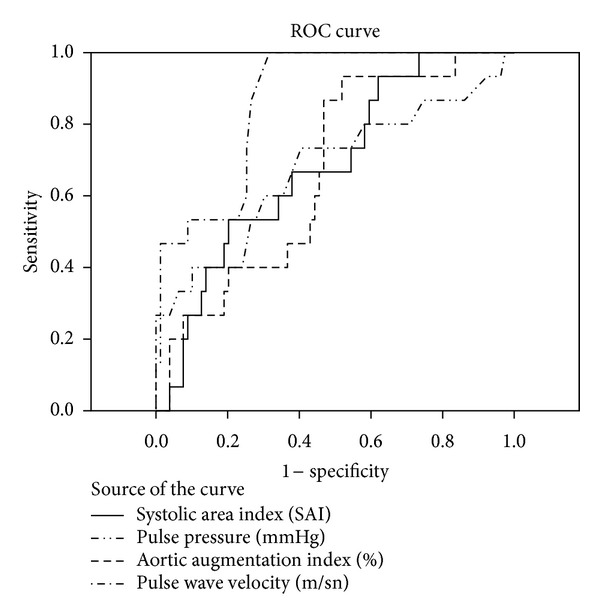
It shows sensitivity and specificity of the parameters.

In comparison PP, AIaortic, RT, DAI, SAI, DRA, and HDL-C were associated with the occurrence of all CV events in univariate analysis, but not after adjustment for other risk factors. Cutoff points for arterial stiffness indices were shown in Table 3. For PWV, a cutoff value of 10.15 was calculated to predict MACE with a sensitivity and specificity of 86.7% and 73.4%, respectively. Sensitivity and specificity of the parameters are seen in the Figures 1 and 2.

## 9. Discussion

As a result of stiffness of arteries afterload increases. It causes the development of left ventricular hypertrophy [14] and oxygen demand [15, 16]. Furthermore, subendocardial ischemia may be triggered [17]. Montalescot et al. [18] examined acute MI patients. Pulse pressure and mean SBP were significantly higher in NSTEMI. In the same study heart rate had a borderline significance between STEMI and NSTEMI patients. However, there is no other study reported which evaluate the arterial stiffness among the types of MI. In our study the incidence of MACE rates and arterial stiffness parameters were similar between MI groups. 

In our study, baseline characteristics and conventional risk factors were similar between groups. We observed the positive and independent association between one-year event rate in AMI patients and arterial stiffness parameters by noninvasive method. 

Recently, it has been shown that the role of PWV was an independent predictor of cardiovascular outcomes. Owing to lack of classical risk factors predicting CV outcomes, PWV seems a powerful decisive parameter [19]. Blacher et al. [20] showed that in 242 patients with end-stage renal disease undergoing hemodialysis, aortic PWV was a strong and independent predictor of cardiovascular events over and above conventional CV risk factors. They defined that PWV ≥ 13.5 m/s was a strong predictor of CV mortality. Vlachopoulos et al. [21] proved the value of PWV in predicting CV events more than conventional risk factors in high risk population (hypertension, coronary heart disease). Total CV events, CV mortality, and all-cause mortality were increased (2.26-, 2.02-, and 1.90-fold, resp.) for per 1 m/s increase in PWV. The authors also showed that aortic PWV analysis provided discriminatory prognostic power in hypertensive [7], diabetic [12], and geriatric [22] population.

 Boutouyrie et al. [13] investigated 1045 essential hypertensive patients without prior CV events or symptoms and observed 97 fatal and nonfatal CV events during 5.7-year followup. MACE observed group had a higher PWV compared to the event-free group. Our findings were also compatible with their findings. Their relative risk for per 3.5 m/s increase in PWV was 1.41 for CV events. In our study, 2.566 (95% CI, 1.447 to 4.551) for per 1 m/s increase in PWV was discriminatory predicting recurrent events. Our relative risk was higher compared to Boutouyrie's results. This probably might be due to the patients characteristics in our group. Anderson et al. [23] reported a cutoff point of 10,6 m/s for PWV in predicting CV events. We found 10.15 m/s with a sensitivity and specificity of 86.7% and 73.4%, respectively.

Pannier et al. [24] examined the simultaneous PWV measurements of the aorta, brachial, and femoral artery in 305 patients and unequivocally proved that only the PWV measurements on the aorta had a predictive value. We measured PWV only from the aorta. Our findings were compatible with their findings as a prognostic significance for cardiovascular events.

Measurement of arterial stiffness by noninvasiveness, is a valuable method. Arteriograph is considerably a tight relationship with the cardiac catheterization measurements [25]. Nevertheless, a study comparing other devices which can measure PWV showed similar PWV values obtained using SphygmoCor (8.1 ± 1.1 m/s) or Arteriograph (8.6 ± 1.3 m/s). However, for Complior method (10.1 ± 1.7 m/s) values were significantly different, because the recorded travel distance for PWV is higher than others [26]. 

## 10. Limitations

Due to the lack of control group, we did not compare MI patients with healthy population. The insufficient number of patients was another deficiency to obtain objective data. In admission and follow-up treatment protocols, revascularization strategies were not compared between groups. Therefore, effectiveness of treatment was not evaluated. Followup was done mainly by telephone in many patients. Hence, the variation of arterial stiffness parameters could not be obtained periodically during followup. 

## 11. Conclusion

The importance of arterial stiffness has been shown for CV events by recent studies. However, further studies for MI patients are required. Patients who experienced MI are potentially at risk and need close followup. In conclusion, evaluation of heart rate, ejection fraction, and arterial stiffness are useful and reliable for predicting recurrent cardiovascular events in patients with AMI. The “gold standard” marker is PWV. PWV is a surrogate measure of arterial stiffness and is not negligible predicting CV outcomes. 

## Figures and Tables

**Table 1:** Hemodynamic variables by the types of MI.

	STEMI (*N* = 45)	NSTEMI (*N* = 49)	*P *
SBP (mmHg)	134.4 ± 21.0	14.0 ± 22.7	NS
DBP (mmHg)	80.9 ± 14.2	79.4 ± 14.8	NS
Heart rate (beat/min)	81 ± 17.7	82.9 ± 11.7	NS
MAP (mmHg)	98.7 ± 15.8	99.6 ± 16.9	NS
PP (mmHg)	53.4 ± 12.0	60.5 ± 12.9	0.007
PWV (m/s)	9.7 ± 2.09	10.2 ± 2.0	NS
AIaortic (%)	19.3 ± 11.6	21.9 ± 13.5	NS
AIbrachial (%)	−29.5 ± 28.6	−32.6 ± 26.0	NS
RT (ms)	110.6 ± 22.9	110.8 ± 24.6	NS
SAI [%]	50.9 ± 7.9	50.0 ± 8.4	NS
DAI [%]	48.8 ± 7.9	50.2 ± 8.5	NS
DRA	50.7 ± 14.0	51.4 ± 14.6	NS

SBP: systolic blood pressure; DBP: diastolic blood pressure; MAP: mean arterial pressure; PP: pulse pressure; PWV: aortic pulse wave velocity; AI: augmentation index; RT: return time; SAI: systolic area index; DAI: diastolic area index; DRA: diastolic reflection area; NS: not significant.

**Table 2:** Clinical, laboratory, and hemodynamic characteristics of the study population.

	Group 1 (*N* = 15)	Group 2 (*N* = 79)	*P *
Age	63.27 ± 14.46	59.97 ± 10.62	NS
Sex			
Male	11 (73%)	61 (77%)	NS
Female	4 (27%)	18 (23%)	
BMI(kg/m^2^)	27.37 ± 4.13	28.31 ± 4.34	NS
Weight (kg)	79.67 ± 13.52	80.58 ± 13.3	NS
Height (m)	1.7 ± 0.05	1.69 ± 0.07	NS
Diabetes mellitus	7(47%)	25 (32%)	NS
Hypertension	10 (67%)	36 (46%)	NS
Hyperlipidemia	9 (60%)	35 (44%)	NS
Family history	8 (53%)	22 (28%)	NS
Smoker	9 (60%)	38 (48%)	NS
SBP (mmHg)	146.8 ± 26.6	135.5 ± 20.7	NS
DBP (mmHg)	82 ± 14.2	79.8 ± 14.6	NS
Heart rate (beat/min)	89.9 ± 15.3	80.4 ± 14.4	0.024
MAP (mmHg)	103.6 ± 17.6	98.37 ± 16.0	NS
PP (mmHg)	64.8 ± 16.1	55.7 ± 11.8	0.012
PWV (m/s)	12.6 ± 2.8	9.4 ± 1.4	<0.001
AIaortic(%)	27.0 ± 10.8	19.4 ± 12.6	0.033
AIbrachial(%)	−20.9 ± 22.1	−33.0 ± 27.7	NS
RT (ms)	84.0 ± 15.7	115.8 ± 21.5	<0.001
SAI [%]	54.5 ± 6.9	49.7 ± 8.1	0.035
DAI [%]	45.4 ± 6.9	50.3 ± 8.2	0.034
DRA	42.7 ± 15.1	52.6 ± 13.6	0.013
eGFR (mL/min/1.73 m^2^)	133.5 ± 46.2	149.2 ± 48.2	NS
HDL	30.1 ± 4.8	35.7 ± 10.1	0.002
EF (%)	45.6 ± 8.9	53.7 ± 9.1	0.002

BMI: body mass index; jugsy: jugulum-symphysis; SBP: systolic blood pressure; DBP: diastolic blood pressure; MAP: mean arterial pressure; PP: pulse pressure; eGFR: estimated glomerular filtration rate; HDL: high density lipoprotein; RT: return time; SAI: systolic area index; DAI: diastolic area index; DRA: diastolic reflection area; PWV: aortic pulse wave velocity; AI: augmentation index; SAI: systolic area index; DAI: diastolic area index; RT: return time; DRA: diastolic reflex area; NS: not significant; EF: ejection fraction; NS: not significant.

**Table 3:** Area values (AUC) and cutoff points after ROC analysis.

Measurements	AUC	Cutoff	Sensitivity	Specificity
PWV (m/s)	0.868	10.1	86.7	73.4
SAI (%)	0.684	52.2	66.7	62
PP (mmHg)	0.674	59.5	73.3	59.5
AIaortic (% )	0.664	21.7	66.7	54.4

PWV: aortic pulse wave velocity; SAI: systolic area index; PP: pulse pressure; AI: augmentation index.
